# A gene prognostic index from cellular senescence predicting metastasis and radioresistance for prostate cancer

**DOI:** 10.1186/s12967-022-03459-8

**Published:** 2022-06-03

**Authors:** Dechao Feng, Dengxiong Li, Xu Shi, Qiao Xiong, Facai Zhang, Qiang Wei, Lu Yang

**Affiliations:** Department of Urology, Institute of Urology, West China Hospital, Sichuan University, Guoxue Xiang #37, Chengdu, 610041 Sichuan People’s Republic of China

**Keywords:** Cellular senescence, Prognostic index, Prostate cancer, Tumor immune microenvironment, Metastasis-free survival, Radioresistance, Immune checkpoint

## Abstract

**Background:**

Senescent cells have been identified in the aging prostate, and the senescence-associated secretory phenotype might be linked to prostate cancer (PCa). Thus, we established a cellular senescence-related gene prognostic index (CSGPI) to predict metastasis and radioresistance in PCa.

**Methods:**

We used Lasso and Cox regression analysis to establish the CSGPI. Clinical correlation, external validation, functional enrichment analysis, drug and cell line analysis, and tumor immune environment analysis were conducted. All analyses were conducted with R version 3.6.3 and its suitable packages.

**Results:**

We used ALCAM and ALDH2 to establish the CSGPI risk score. High-risk patients experienced a higher risk of metastasis than their counterparts (HR: 10.37, 95% CI 4.50–23.93, p < 0.001), consistent with the results in the TCGA database (HR: 1.60, 95% CI 1.03–2.47, p = 0.038). Furthermore, CSGPI had high diagnostic accuracy distinguishing radioresistance from no radioresistance (AUC: 0.938, 95% CI 0.834–1.000). GSEA showed that high-risk patients were highly associated with apoptosis, cell cycle, ribosome, base excision repair, aminoacyl-tRNA biosynthesis, and mismatch repair. For immune checkpoint analysis, we found that PDCD1LG2 and CD226 were expressed at significantly higher levels in patients with metastasis than in those without metastasis. In addition, higher expression of CD226 significantly increased the risk of metastasis (HR: 3.65, 95% CI 1.58–8.42, p = 0.006). We observed that AZD7762, PHA-793887, PI-103, and SNX-2112 might be sensitive to ALDH2 and ALCAM, and PC3 could be the potential cell line used to investigate the interaction among ALDH2, ALCAM, and the above drugs.

**Conclusions:**

We found that CSGPI might serve as an effective biomarker predicting metastasis probability and radioresistance for PCa and proposed that immune evasion was involved in the process of PCa metastasis.

## Introduction

The world is now in an era of an aging population, which contributed 16% of cancer cases between 2005 and 2015 [1]. By 2030, approximately 20% of the world’s population will be aged 65 or older, with an exponential augmentation in the prevalence of prostate cancer (PCa) because this disease is most common in men 65 and older [2, 3]. Moreover, the prevalence of metabolic disorders apparently increases and further facilitates the morbidity and mortality of PCa [4, 5]. PCa has the highest morbidity and mortality among urothelial malignancies, with an estimated 1.4 million new male cases and 375,000 deaths worldwide in 2020 [6]. Thus, with population aging and increased life expectancy globally, the improvement of prognosis is an increasingly important area for PCa patients.

PCa could be a pernicious disease for patients with intermediate- or high-risk localized and locally advanced cancer who ask for local curative treatment in most cases [7]. The curative treatments of PCa include radical prostatectomy, intensity-modulated radiotherapy, and proton beam therapy with or without hormone therapy [8, 9]. Unfortunately, approximately one-third of such patients develop this disease due to metastasis and radiotherapy resistance [10]. Cellular senescence is a predominant trait of aged organisms, and excessive accumulation of senescent cells in tissues can contribute to the onset and progression of various age-related diseases, including cancer [11, 12]. The features of the senescence phenotype usually consist of the activation of a chronic DNA damage response, the involvement of various cyclin-dependent kinase inhibitors, increased secretion of proinflammatory and tissue-remodeling factors, induction of antiapoptotic genes, altered metabolic rates, and endoplasmic reticulum stress [13]. To date, the extent to which cellular senescence contributes to PCa remains elusive. In view of this, we developed and validated a cellular senescence-related gene prognostic index (CSGPI) to predict metastasis and tumor radioresistance and explored the related changes in the tumor immune microenvironment (TME) for PCa patients undergoing radical radiotherapy.

## Methods

### Data sources and clinical analysis

**Fig. 1:**
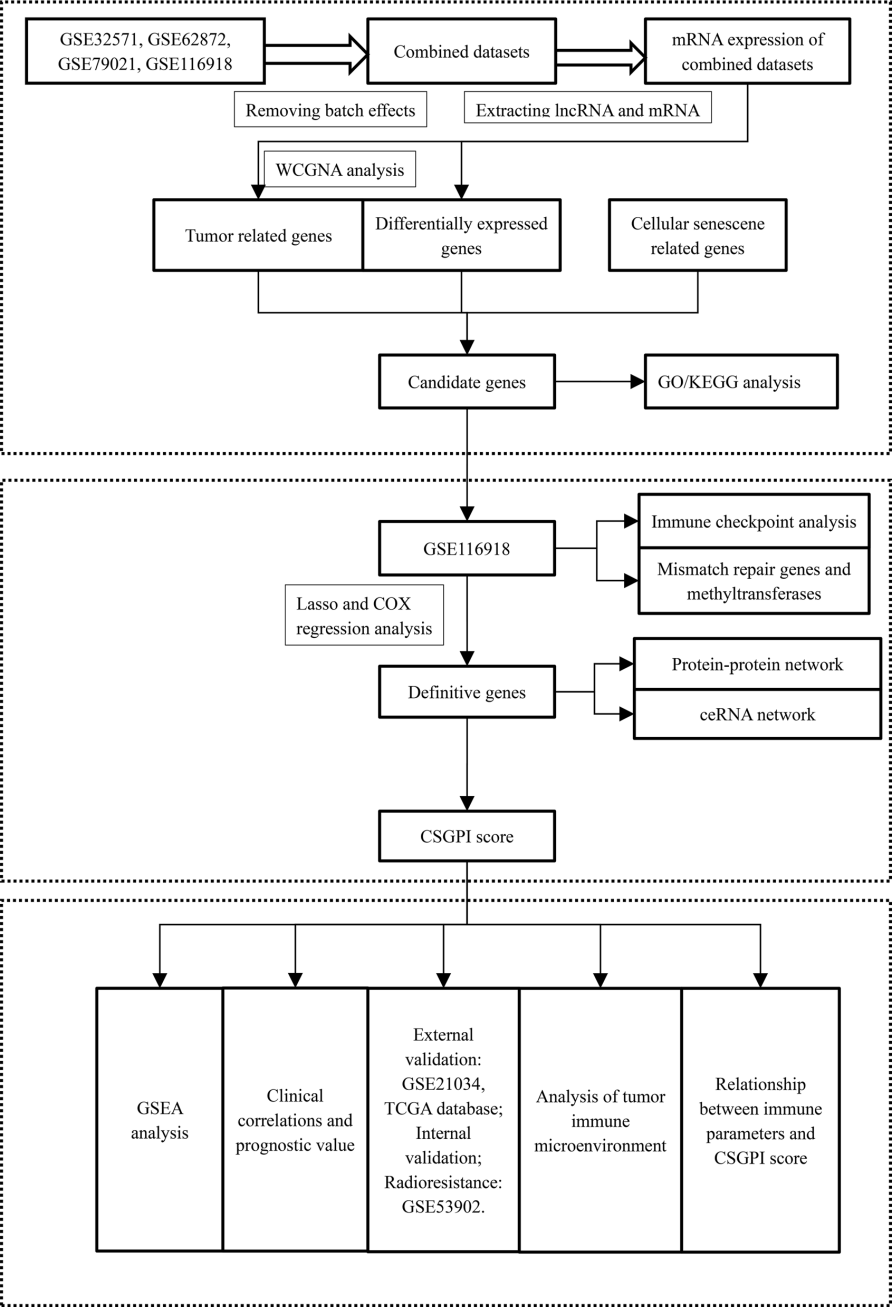
The flowchart of this study. *WGCNA* weighted gene coexpression network analysis; *GO* gene ontology; *KEGG* Kyoto Encyclopedia of Genes and Genome; *GSEA* gene set enrichment analysis; *CSGPI* cellular senescence-related gene prognostic index; *mRNA* message RNA; *lncRNA* long noncoding RNA

Our study has been registered in the ISRCTN registry (No. ISRCTN11560295). In total, we obtained GSE32571 [14], GSE62872 [15], GSE79021 [16], and GSE116918 [17] from the Gene Expression Omnibus (http://www.ncibi.nlm.nih.gov/geo/) [18] to develop CSGPIs related to metastasis. The specific process of combing GEO datasets could be seen in our previous study [19]. The R package “inSilicoMerging” and the “removeBatchEffect” function of R pachkage “limma” were adopted to merge the four datasets and to further remove the batch effect, respectively [20]. Prostate adenocarcinoma data in the TCGA database and GSE21034 [21] were used for external validation of the prognostic value of the CSGPI. Seventy percent of patients in GSE116918 [17] were extracted randomly to internally validate the prognostic value of the CSGPI. Moreover, radiotherapy resistance was tested using GSE53902 [22]. GSE32571 [14], GSE62872 [15], and GSE79021 [16] were used to identify differentially expressed genes (DEGs) and tumor-related genes using weighted gene coexpression network analysis (WGCNA). DEGs were defined by llogFCl ≥ 0.4 and p.adj. < 0.05. Tumor-related genes were defined by a coefficient ≥ 0.3 and p.adj. < 0.001. Cellular senescence-related genes were obtained from the GeneCards database [23]. Subsequently, we performed an intersection of tumor-related genes, DEGs, and cellular senescence-related genes to determine the candidate genes (Fig. 1). A total of 248 samples with complete clinical data in GSE116918 [17] were used to determine the definitive genes through Lasso and Cox regression analysis (Fig. 1). Thereafter, we established a formula for risk stratification: CSGPI risk score = 0.97428*ALCAM-0.85073*ALDH2. We divided the patients into high- and low-risk groups based on the median CSGPI risk score and tested its clinical correlation and prognostic value for metastasis-free survival (MFS) in GSE116918 [17]. External validation using the TCGA database, GSE21034 [21] and GSE53902 [22], and internal validation were also conducted (Fig. 1). In addition, to further determine the prognostic role of the CSGPI risk score for PCa patients, we stratified the 248 patients according to the latest European Association of Urology (EAU) and National Comprehensive Cancer Network (NCCN) guidelines [24, 25] and compared these factors using Cox regression analysis in terms of MFS.

### Protein–protein interaction and competing endogenous RNA (ceRNA) network

We used the GeneMANIA [26] database to explore the possible links of gene interactions. In addition, we determined the long noncoding RNAs (lncRNAs) that were differentially expressed and associated with MFS, and we further established the ceRNA network according to LncBase [27] and the miWalk database [28].

### Functional enrichment analysis

Gene Ontology (GO) referred to three aspects as follows: biological process, cell composition, and molecular function. We conducted GO and Kyoto Encyclopedia of Genes and Genome (KEGG) analyses to explore the potential biological functions and signaling pathways of the candidate genes using the package R “clusterProfiler”. We divided the patients in GSE116918 [17] into high- and low-risk groups based on the median CSGPI risk score. Subsequently, gene set enrichment analysis (GSEA) was performed using GSEA software (version 3.0) (http://www.gsea-msigdb.org) [29]. “h.all.v7.4.symbols.gmt” and “c2.cp.kegg.v7.4.symbols.gmt” from the molecular signature database [30] were used to detect pathways and molecular mechanisms. Considering the gene expression profile and risk groups, the minimum gene set was 5, and the maximum was 5000. p < 0.05 and false discovery rate < 0.25 were considered statistically significant.

### DNA mismatch repair (MMR) gene mutation and DNA methylation analysis

The MMR genes and methyltransferases were obtained from a previous study [31]. We analyzed the correlation between these genes and the CSGPI score through Spearman analysis.

### TME, drug, and cell line analysis

Seventeen common immune checkpoint genes were used for the analysis in this study. In addition, we used the Xcell [32] algorithm to analyze the TME through the package R “IOBR” [33]. Differential expression and Spearman analyses of these parameters were performed. We analyzed the drug sensitivity of ALCAM and ALDH2 through GSCALite [34]. Subsequently, we analyzed PCa-related cell lines of ALCAM and ALDH2 and the possible sensitive drugs through the canSAR database [35].

### Statistical analysis

All analyses were conducted with R version 3.6.3 and its suitable packages. Cytoscape 3.8.2 [36] was used to establish the ceRNA network. Normality tests were performed using the Shapiro–Wilk method, and when the sample did not conform to a normal distribution, Spearman’s correlation analysis was conducted to describe the correlation between quantitative variables. The significance of two groups of samples was tested by the Wilcoxon test and Kruskal–Wallis test for three or more groups. We also carried out survival analysis using the log rank test. Only variables that were statistically significant in the univariable Cox regression analysis were included in the multivariable Cox regression models. ROC curves were generated using the R packages “timeROC” and “pROC”. Each outcome was regarded as statistically significant with a two-sided p value of < 0.05. Significant mark: ns, p ≥ 0.05; *, p < 0.05; **, p < 0.01; ***, p < 0.001.

## Results

### Development of CSGPI and its clinical value

**Fig. 2:**
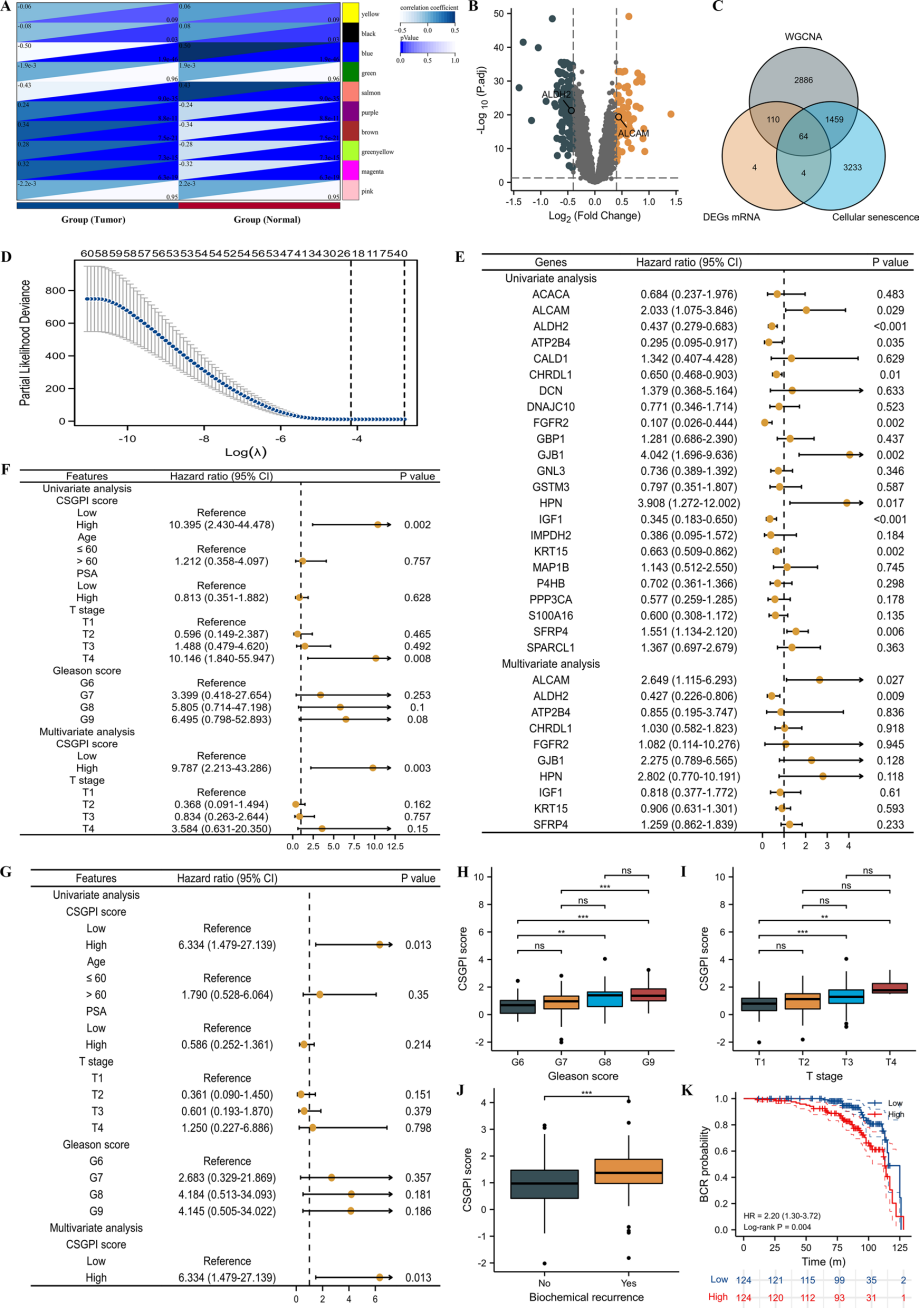
The screening process of definitive genes and baseline features. **A** modules and phenotype; **B** volcano plot; **C** venn diagram; **D** gene screening of Lasso regression; **E** univariate and multivariate Cox analysis of candidate genes; **F** univariate and multivariate Cox analysis of CSGPI score and clinical parameters for metastasis-free survival; **G** univariate and multivariate Cox analysis of CSGPI score and clinical parameters for metastasis-free survival after biochemical recurrence; **H** comparison between Gleason score and CSGPI score; **I** comparison between T stage and CSGPI score; **J** comparison between biochemical recurrence and no biochemical recurrence for CSGP score; **K** Kaplan–Meier curve of probability of biochemical recurrence. *CSGPI* cellular senescence-related gene prognostic index

We detected 64 candidate genes through the intersection of tumor-related genes, DEGs, and cellular senescence-related genes (Fig. 2A–C). A total of 248 tumor samples in GSE116918 [17] were used to identify prognostic genes. Subsequently, we identified ALCAM and ALDH2 as independent prognostic genes through Lasso and Cox regression analyses (Fig. 2D, E) and further established the CSGPI score based on the following formula: CSGPI risk score = 0.97428*ALCAM-0.85073*ALDH2. The Sanky plot showed clinical indicators and CSGPI scores. We divided the patients into high- and low-risk groups according to the median CSGPI score. We confirmed that the CSGPI score could be used as an independent risk factor for metastasis-free survival (MFS) (HR: 9.787, 95% CI 2.213–43.286, p = 0.003; Fig. 2F) and MFS after biochemical recurrence (BCR) (HR: 6.334, 95% CI 1.479–27.139, p = 0.013; Fig. 2G). Furthermore, the prognostic role of the CSGPI was confirmed again when compared to the EAU and NCCN risk groups. In addition, we observed an increasing trend in the CSGPI score with increasing Gleason score (Fig. 2H), T stage (Fig. 2I), and the presence of BCR (Fig. 2J). Furthermore, patients in the high-risk group had a higher risk of BCR than those in the low-risk group (HR: 2.20, 95% CI 1.30–3.72, p = 0.004; Fig. 2K).

**Fig. 3:**
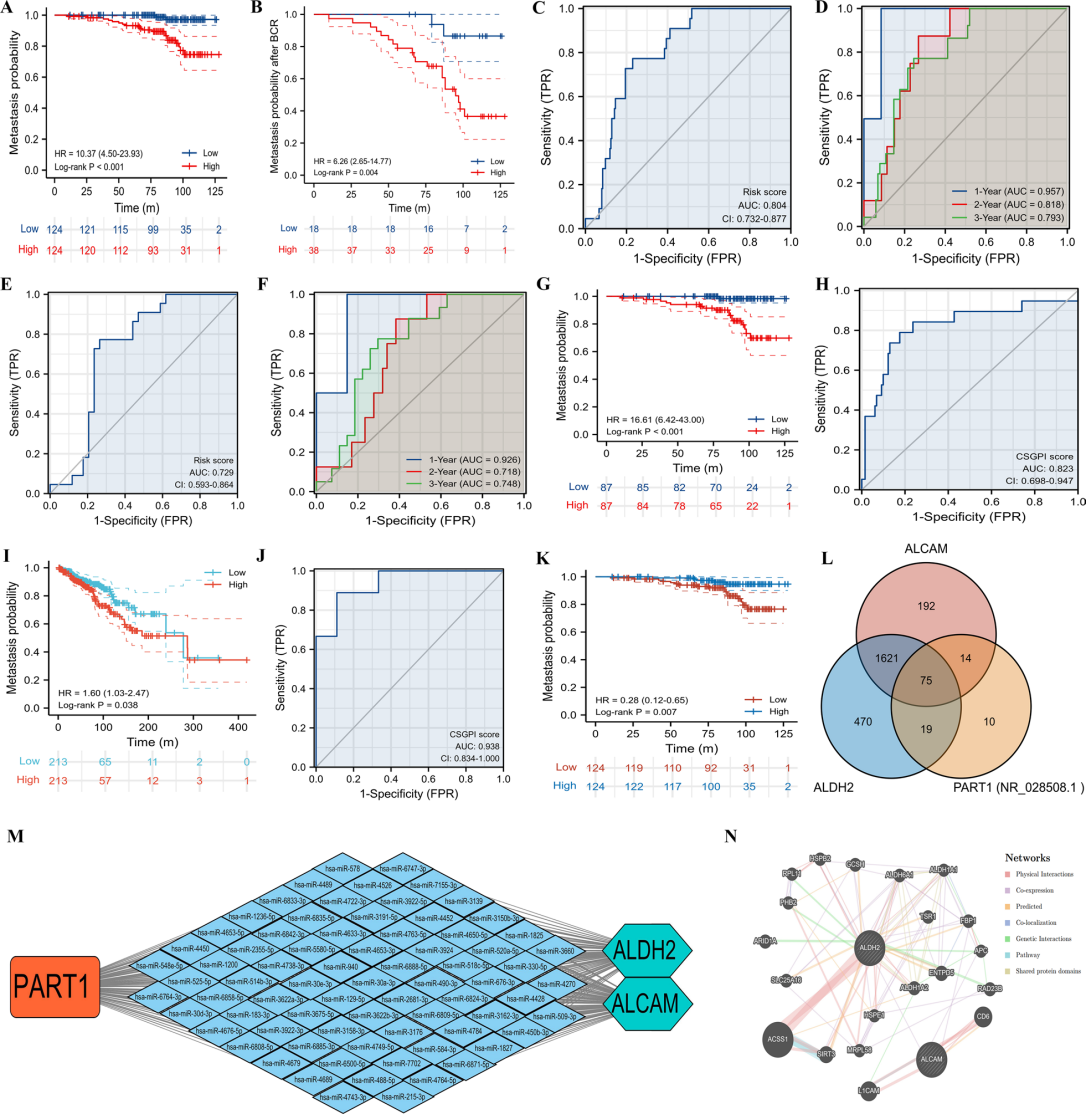
Clinical values and interaction networks. **A** Kaplan–Meier curve of metastasis-free survival; **B** Kaplan–Meier curve of metastasis-free survival after biochemical recurrence; **C** ROC curve of CSGPI score for metastasis; **D** time-dependent ROC curve of CSGPI score for metastasis; **E** ROC curve of CSGPI score for metastasis after biochemical recurrence; **F** time-dependent ROC curve of CSGPI score for metastasis after biochemical recurrence; **G** Kaplan–Meier curve of metastasis-free survival in internal validation; **H** ROC curve of CSGPI score for metastasis using GSE21034 [21]; **I** Kaplan–Meier curve of metastasis-free survival in TCGA database; **J** ROC curve of CSGPI score for radioresistance; **K** Kaplan–Meier curve of metastasis-free survival in terms of lncRNA PART1; **L** Venn plot of miRNA intersection of ALCAM, ALDH2, and PART1; **M** interaction network of competing endogenous RNAs; **N** protein–protein interaction network. *ROC* receiver operating characteristic; *CSGPI* cellular senescence-related gene prognostic index

We observed that high-risk patients experienced a higher risk of metastasis than their counterparts regardless of MFS (HR: 10.37, 95% CI 4.50–23.93, p < 0.001; Fig. 3A) or MFS after BCR (HR: 6.26, 95% CI 2.65–14.77, p = 0.004; Fig. 3B). The CSGPI score had better diagnostic accuracy for MFS (Fig. 3C, D) and MFS after BCR (Fig. 3E, F). In terms of internal validation, we observed a similar result for MFS (HR: 16.61, 95% CI 6.42–43.00, p < 0.001; Fig. 3G). For external validation of diagnosis, we also detected a consistent outcome using GSE21034 [21] (AUC: 0.823, 95% CI 0.698–0.947; Fig. 3H). For prostate adenocarcinoma in the TCGA database, high-risk patients had a higher risk of metastasis than low-risk patients (HR: 1.60, 95% CI 1.03–2.47, p = 0.038; Fig. 3I). Furthermore, CSGPI had high diagnostic accuracy distinguishing radioresistance from no radioresistance (AUC: 0.938, 95% CI 0.834–1.000; Fig. 3J). We detected that the long noncoding RNA (lncRNA) PART1 was closely related to MFS in the high- and low-risk groups (HR: 0.28, 95% CI 0.12–0.65%; Fig. 3K). Subsequently, we found that lncRNA PART1 might modulate the expression of ALCAM and ALDH2 through interaction with 75 possible miRNAs (Fig. 3L) and further established the ceRNA network (Fig. 3M). Moreover, ALDH2 and ALCAM might interact through coexpression and genetic interactions (Fig. 3N).

### Functional analysis

**Fig. 4:**
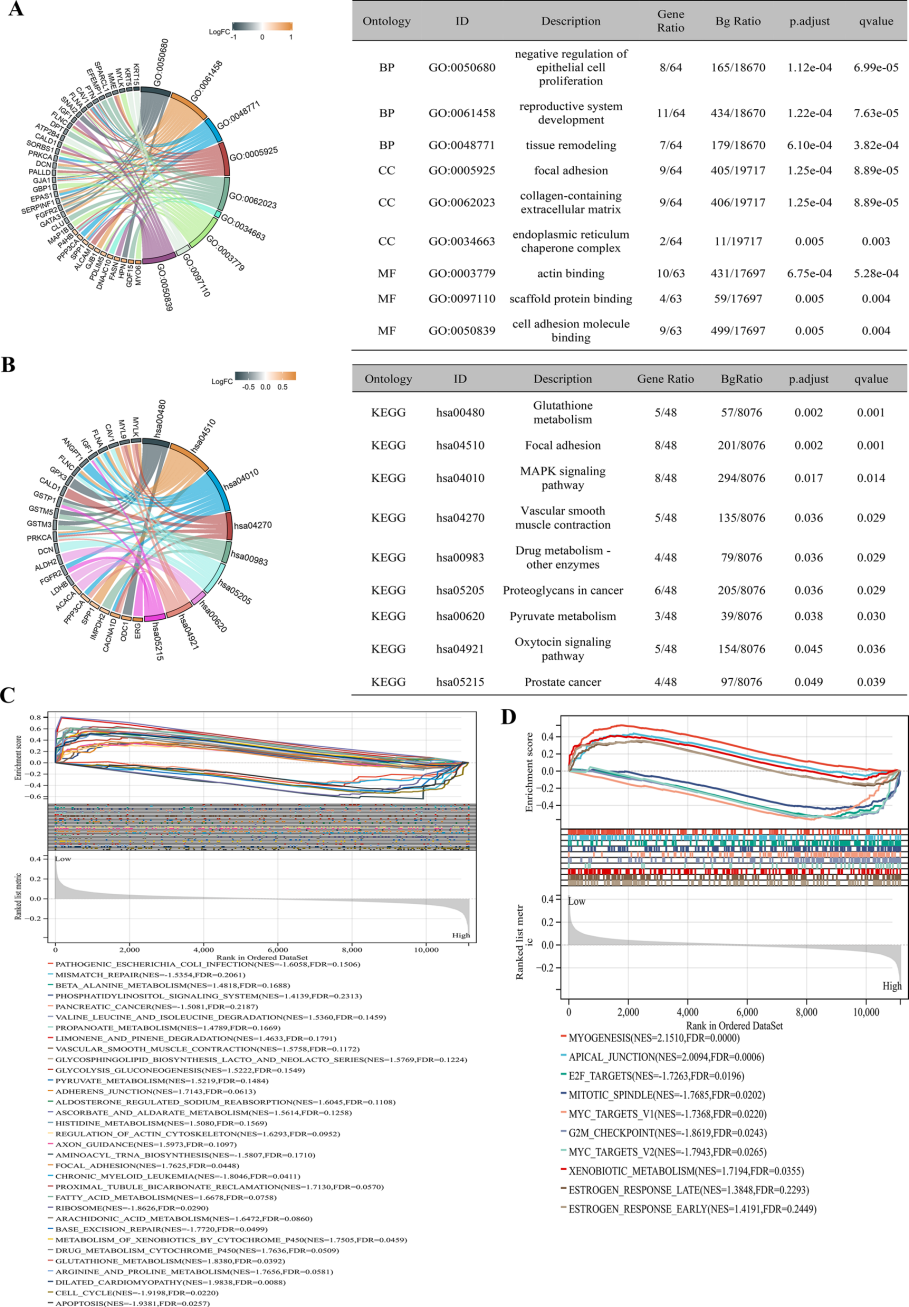
Functional enrichment analysis. **A** GO analysis; **B** KEGG analysis; **C** GSEA C2 analysis; **D** GSEA hallmark analysis. *GO* Gene Ontology; *KEGG* Kyoto Encyclopedia of Genes and Genome; *GSEA* gene set enrichment analysis

GO analysis showed that the 64 candidate genes might engage in the process of negative regulation of epithelial cell proliferation, reproductive system development, tissue remodeling, focal adhesion, collagen-containing extracellular matrix, endoplasmic reticulum chaperone complex, actin binding, scaffold protein binding, and cell adhesion molecule binding (Fig. 4A). KEGG analysis indicated that these genes might be involved in glutathione metabolism, focal adhesion, the MAPK signaling pathway, vascular smooth muscle contraction, drug metabolism, proteoglycans in cancer, pyruvate metabolism, the oxytocin signaling pathway, and PCa (Fig. 4B). GSEA showed that high-risk patients were highly associated with apoptosis, cell cycle, ribosome, base excision repair, aminoacyl-tRNA biosynthesis, and mismatch repair, while low-risk patients were closely associated with glutathione metabolism, arginine and proline metabolism, drug metabolism cytochrome P450, focal adhesion, fatty acid metabolism, arachidonic acid metabolism, regulation of actin cytoskeleton, and so on (Fig. 4C). For the hallmarks, high-risk patients were closely associated with E2F targets (genes encoding cell cycle-related targets of E2F transcription factors), mitotic spindle (genes important for mitotic spindle assembly), MYC targets V1, G2 M checkpoint (genes involved in the G2/M checkpoint, as in progression through the cell division cycle), and MYC targets V2, whereas the low-risk patients were enriched in myogenesis and apical junction (genes encoding components of apical junction complex.), xenobiotic metabolism (genes encoding proteins involved in the processing of drugs and other xenobiotics), estrogen response late, and estrogen response early (Fig. 4D).

### TME, drug, and cell line analysis

**Fig. 5:**
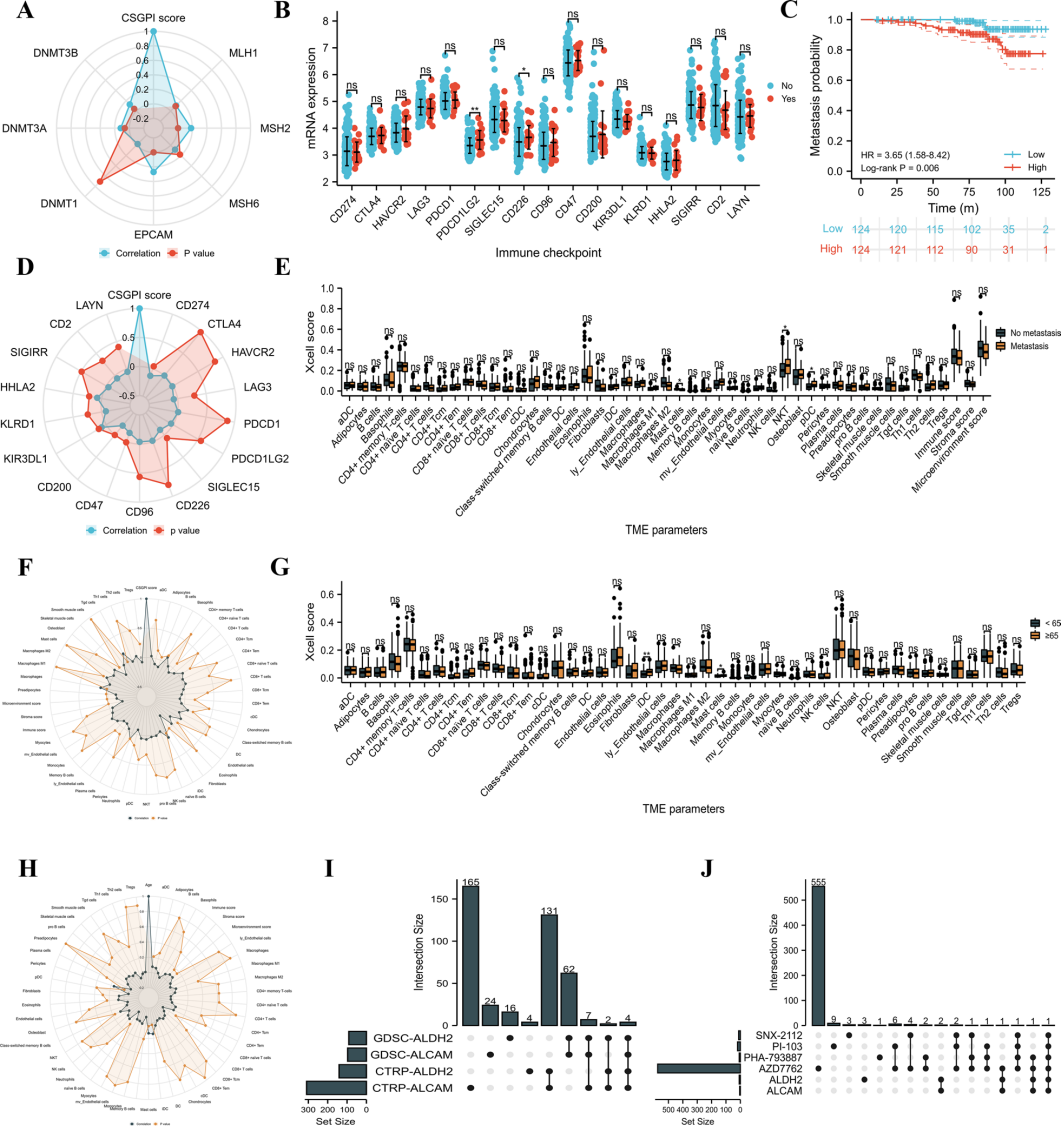
Drug, cell line, and TME analysis. **A** Radar plot showing the correlation between the CSGPI score and mismatch repair genes and methyltransferases; **B** comparison between the metastasis and no metastasis groups concerning immune checkpoint genes; **C** Kaplan–Meier curve of metastasis-free survival for CD226; **D** radar plot showing the correlation between the CSGPI score and immune checkpoint genes; **E** comparison between the metastasis and no metastasis groups for TME parameters; **F** radar plot showing the correlation between the CSGPI score and TME parameters; **G** comparison between the ≥ 65 and < 65 groups for TME parameters; **H** radar plot showing the correlation between age and TME parameters; **I** upset plot of commonly sensitive drugs of ALCAM and ALDH2; **B** upset plot of common cell lines of ALCAM, ALDH2, and sensitive drugs. *GDSC* genomics of drug sensitivity in cancer; *CTRP* the cancer therapeutics response portal; *TME* tumor immune microenvironment; *CSGPI* cellular senescence-related gene prognostic index

The MMR gene and methyltransferase analyses showed that the CSGPI score was positively related to MSH2 (r: 0.18), EPCAM (r: 0.27), and DNMT3B (r: 0.13) (Fig. 5A). For immune checkpoint analysis, we found that PDCD1LG2 and CD226 were expressed at significantly higher levels in patients with metastasis than in those without metastasis (Fig. 5B). In addition, higher expression of CD226 significantly increased the risk of metastasis (HR: 3.65, 95% CI 1.58–8.42, p = 0.006; Fig. 5C). Spearman analysis showed a negative correlation between the CSGPI score and CD274 (r: − 0.13), CD47 (r: − 0.16) and CD200 (− 0.21) (Fig. 5D). Compared to patients in the no metastasis group, patients in the metastasis group scored significantly higher for natural killer T cells (p = 0.046) and plasmacytoid dendritic cells (p = 0.041) but scored lower for mast cells (p = 0.03) (Fig. 5E). Spearman analysis showed that CSGPI was positively associated with immature dendritic cells (r: 0.13, p = 0.045), gamma delta T cells (Tgd) (r: 0.17, p = 0.008), and macrophages (r: 0.14, p = 0.030) but negatively related to endothelial cells (r: − 0.14, p = 0.032), preadipocytes (r: − 0.14, p = 0.030), skeletal muscle cells (r: − 0.15, p = 0.012), stromal score (r: − 0.24, p < 0.001), and microenvironment score (r: − 0.15, p = 0.019) (Fig. 5F). In addition, patients aged and over 65 years scored significantly higher in terms of immature dendritic cells (p = 0.01) and mast cells (p = 0.019) than their counterparts (Fig. 5G). Spearman analysis showed that age was significantly associated with immature dendritic cells (r: 0.14, p = 0.02), mast cells (r: 0.13, p = 0.04), and pericytes (r: 0.16, p = 0.11) (Fig. 5H). We observed that AZD7762, PHA-793887, PI-103, and SNX-2112 might be sensitive to ALDH2 and ALCAM (Fig. 5I), and PC3 could be the potential cell line used to investigate the interaction among ALDH2, ALCAM, and the above drugs (Fig. 5J).

### Discussion

PCa has long been a question of great interest in the field of urology. It is well known that metastatic castration-resistant PCa is the leading cause of death, and the prevalence of metastasis is increasing [37]. With an aging population worldwide, the problem will only grow worse. Prior studies have noted that a doubling time of prostate-specific antigen (PSA) ≤ 7.5 months or PSA ≥ 0.5 ng/mL are independent risk factors for MFS [38]; however, BCR is not a specific indicator of overall survival and PCa-related mortality because a subset of patients only undergo rising PSA levels and will not progress [39].

PCa is an age-related disease, and senescent cells accumulate with age in all tissues. Although senescent cells cannot replicate, these cells are metabolically active and form an inflammatory microenvironment through the senescence-associated secretory phenotype (SASP) [40]. SASP consists of various proinflammatory mediators, including cytokines, chemokines, growth factors and proteases, and enables senescence of adjacent nonsenescent cells through paracrine pathways and further contributes to the inflammatory microenvironment [41]. Many age-related diseases, such as osteoarthritis and atherosclerosis, have been demonstrated to be correlated with cellular senescence and the accumulation of senescent cells [42]. Senescent cells have also been found in the aging prostate, and previous studies indicate that SASP plays an important role in tumorigenesis despite the tumor suppression of senescence [40, 43, 44]. However, few previous studies have developed a simple and practical genetic biomarker to predict metastasis for patients undergoing radical radiotherapy. In this study, we established and validated a CSGPI score with two genes to predict MFS for PCa patients undergoing radical prostatectomy or radiotherapy. The origins of senescence-triggering mechanisms consist of therapy-induced, oncogene-induced and age-induced senescence [40]. In this study, the high diagnostic accuracy of the CSGPI indirectly demonstrated the link between cellular senescence and radioresistance. The proinflammatory process of senescence derives from DNA damage caused by various stimuli and amplifying effects of SASP [40, 45], which was consistent with our findings that high-risk patients were highly enriched in cell cycle and MMR. MSH2 and EPCAM might be involved in the process of metastasis and radioresistance in patients undergoing radiotherapy. Previous studies have reported that several miRNAs, such as miR-30 families, are dysregulated in PCa and interact with the p16INK4A/Rb pathway, which is associated with cellular senescence [40, 46]. Our ceRNA network indicated that hsa-miR-30a-3p, hsa-miR-30d-3p, and hsa-miR-30e-3p might contribute to the process of cellular senescence.

PDCD1LG2 and CD226 showed significantly higher expression in patients with metastasis, and patients with higher expression levels of CD226 had susceptibility to metastasis. In addition, natural killer T cells and plasmacytoid dendritic cells scored significantly higher in the metastasis group than in their counterparts. In the human body, CD226 is highly expressed on the surface of NK cells and CD8 + T cells and can activate the function of these cells [47]. Thus, we proposed that immune evasion might be involved in the process of metastasis, and the positive correlation between macrophages and CSGPI score supported this opinion as well. Immunosenescence, defined as the changes in the immune system associated with age, has been sought to produce a progressive deterioration in the ability to respond to infections and to develop immunity after vaccination, both of which are associated with a higher mortality rate in the elderly [48]. However, our findings indicated that immunosenescence was not necessarily correlated with age in patients with metastatic PCa. Moreover, we must admit the following limitations. First, gene expression signatures are subject to sampling bias caused by intratumor genetic heterogeneity. In addition, the microenvironment features might be distinct in different tumor regions, such as the tumor core and invasive margin. More importantly, all findings, such as the ceRNA network in this study, still warrant further confirmation.

## Conclusions

We found that CSGPI might serve as an effective biomarker predicting metastasis probability and radioresistance for PCa and proposed that immune evasion was involved in the process of PCa metastasis.

## Data Availability

The datasets presented in this study can be found in online repositories. The names of the repository/repositories and accession number(s) can be found in the article.
